# Dexmedetomidine and Tear Production: Evaluation in Dogs as Spontaneous Model for Ocular Surface Disorders

**DOI:** 10.3390/vetsci8020028

**Published:** 2021-02-16

**Authors:** Simona Di Pietro, Claudia Giannetto, Annastella Falcone, Giuseppe Piccione, Fulvio Congiu, Francesco Staffieri, Elisabetta Giudice

**Affiliations:** 1 Department of Veterinary Sciences, University of Messina, Viale Palatucci, 98168 Messina, Italy; claudia.giannetto1@unime.it (C.G.); a_stella@hotmail.it (A.F.); gpiccione@unime.it (G.P.); fulviocongiu@libero.it (F.C.); egiudice@unime.it (E.G.); 2 Department of Emergency and Organ Transplantation, University of Bari, 70124 Bari, Italy; francesco.staffieri@uniba.it

**Keywords:** dexmedetomidine, dog, exposure keratitis, tear production, translational research

## Abstract

**Simple Summary:**

The general anesthesia or sedation reduces both the tear production and the stability of tear film that protect corneal surface, predisposing itself to the exposure keratopathy. The aim of the present study was to evaluate the effects of intramuscular dexmedetomidine (DEX) on canine tear production, measured by standardized Schirmer Tear Test 1 (STT-1) strips, for the 8 h following sedation, in dogs. A significant effect of time on canine tear production was found, highlighting that dexmedetomidine sedative protocol significantly affects tear production in dogs. It is recommended to treat the canine eyes with tear substitutes to protect ocular surface health and the welfare of the dogs. The ocular lubrication should be performed during and up to 12 h after sedation. The present report could provide preliminary information to better understand the effect of DEX on the tear film dynamics.

**Abstract:**

Tear film provides lubrication and protection to the ocular surface. The sedation reduces tear production, often leading to perioperative exposure keratopathy. The aim of the present study was to report the effects of intramuscular dexmedetomidine on canine tear production, measured by STT-1, for an experimental period of 8 h after sedation. Ten dogs who underwent sedation for routine radiologic assessment were recruited for the study. In all animals, tear production in right and left eyes was measured 15 min before sedation (T0: basal values) and 20 min (T20), 1 h (T1), 2 h (T2), 4 h (T4) and 8 h (T8) after drug administration. Analysis of variance and post hoc Bonferroni test (*p* < 0.05) were performed. A significant effect of time on canine tear production was found. The tear production returned to basal values at T8. So, it is recommended to treat the canine eyes with tear substitutes during and up to 12 h after sedation.

## 1. Introduction

The tear film is a thin transparent fluid that covers the ocular surface. It consists of an aqueous/mucin phase and a distinct superficial lipid layer. The latter layer contains many different lipids, including cholesterol, fatty acids and phospholipids, which are mainly secreted by meibomian glands. The most significant role of the lipid layer is to reduce evaporation of tears from the corneal surface. The aqueous layer contains a specific variety of proteins, electrolytes and water secreted from the lacrimal gland and conjunctival epithelium. The mucins are secreted by specialized goblet cells in the conjunctival epithelium. The aqueous/mucin phase provides oxygen and nutrients to the corneal tissue and flushes away epithelial debris, toxins and foreign bodies from the ocular surface, and the mucins help to stabilize the tear film. The tear film provides lubrication and protection to the ocular surface, as well as maintains a smooth, refractive surface for optimal visual performance. 

The balance between the tear production by lacrimal gland and loss via evaporation, absorption and drainage characterizes the dynamic process of tear flow. This process is under the hormonal and autonomic nervous system control and it is influenced by environmental conditions. Blinking also regulates the tear production [1]. 

As water is a major constituent of the eye, ocular physiology and multiple ocular disorders could be affected by changes in hydration status, in particular, dehydration [2]. Recently, ophthalmic research has been focused on aquaporins (AQPs), a family of water channel proteins that controls osmotically driven water and fluid transport across cell membranes. The authors underlined the following functional roles of AQPs in the eye function: water balance maintenance to ensure transparency in cornea and lens, aqueous humor production, corneal wound healing and regulation of the tear film osmolarity and retinal homeostasis [3,4,5]. 

In veterinary medicine, recent interesting investigations regarding canine tears have been reported, such as the presence of AQPs (AQP0, AQP1, AQP3–5 and AQP9) in different canine ocular tissues [6]. The expression of aquaporin-1 (AQP1) by Western blot analysis was found in canine eye tears, suggesting its specific role in tear secretion [7].

Several factors can lead to a loss of homeostasis of the tear film, with subsequent instability and hyperosmolarity, ocular surface inflammation and damage and neurosensory abnormalities. 

The general anesthesia or sedation can cause adverse ocular side effects, such as lagophthalmos, loss of eyelid reflex, decreased basal tear production and reduced stability of tear film protecting the corneal surface [8]. The main cause is the suppression of parasympathetic innervation of the lacrimal gland, as demonstrated in rats and rabbits experimentally para-sympathectomized by sectioning of the greater superficial petrosal nerve [9,10,11]. A marked decrease in tear production after induction of anesthesia has been reported in humans [12,13,14,15] and in dogs, using different anesthetic and sedative protocols [16,17,18,19,20,21]. The loss of corneal integrity resulting from perioperative dry eye can lead to corneal abrasions, which are the most common ocular complication in humans during general anesthesia for non-ophthalmic surgery [20,22]. Post-anesthesia corneal lesions have been reported also in dogs [17,20,21], cats [23,24] and horses [25].

Over the last few years, several investigations have integrated companion animals into preclinical studies to expand the knowledge gained from studies in other animal models and generate discoveries that will benefit the health of humans and animals [26]. Some authors reported the benefits of using dogs for translational research in different biomedical fields such as oncology [27] and neurology [28]. In ophthalmology, the canine ocular anatomy is more similar to humans than small laboratory animals and some spontaneously occurring ocular surface diseases are comparable in both canine and human species [29]. In contrast, naturally acquired ocular surface pathology is much less common in rabbits [30] and is rare in mice and rats [31]. 

Corneal injury due to trauma, surgery or dry eye is common in human and veterinary patients, and the resulting corneal damage remains one of the leading causes of blindness in animals and people worldwide [32]. 

Exposure keratopathy is an ocular complication of the perioperative reduction of tear secretion due to sedation or general anesthesia in both humans and animals [8,33]. 

Currently, there is increased interest in the use of dexmedetomidine as an agent for procedural sedation. Dexmedetomidine (DEX) is a α2-adrenergic agonist with beneficial sedative properties and a limited adverse effect profile. Dexmedetomidine causes its physiologic effects by activation of specific transmembrane α2 adrenergic receptors at various locations throughout the central nervous system. These effects include sedation, anxiolysis and analgesia. 

Moreover, dexmedetomidine was approved by the Food and Drug Administration for sedation of adults requiring mechanical ventilation for up to 24 h and for monitored anesthesia care or procedural sedation occurring within the operating room [34,35].

In veterinary medicine, previous studies have been performed to evaluate the effect of dexmedetomidine in dogs alone or in combination with butorphanol, methadone, morphine or tramadol [36,37]. The sedative and analgesic drug was used in dogs and cats to perform clinical procedures, minor surgical and dental procedures and as a pre-anesthetic prior to general anesthesia. 

Some authors reported the effect of dexmedetomidine-butorphanol combination on canine tear production. They showed that Schirmer Tear Test 1 (STT-1) readings were significantly lower than basal levels even 15 min after reversal of dexmedetomidine by atipamezole [18]. For this purpose, the aim of the present study was to report the effects of intramuscular dexmedetomidine on canine tear production, measured by STT 1 for an experimental period of 8 h after sedation.

## 2. Materials and Methods

### 2.1. Animals and Sampling Protocol

Ten client-owned dogs who underwent sedation for routine radiologic assessment were recruited for the study after the owners’ written consent was obtained. 

All treatments, housing and animal care reported in this study were configured as a veterinary medical diagnostic clinical trial and were carried out in accordance with the EU Directive 2010/63/EU for animal experiments. 

Prior to the study, each animal underwent a complete physical examination and laboratory tests, including complete blood counts (CBC) and biochemical profile (urea, creatinine, total protein and albumin). An ocular examination including indirect ophthalmoscopy, slit-lamp biomicroscopy, STT-1 and fluorescein staining of both eyes was also performed. 

Inclusion criteria were: dogs not belonging to brachycephalic breeds, corresponding to a physical status I or II according to the American Society of Anesthesiologists (ASA) classification and unaffected by systemic or ocular disease that could affect STT-1 results. Exclusion criteria included a baseline STT-1 reading of less than 10 mm/min and a clinical and laboratory evidence of hydration status disorders. In all animals, the STT-1 tear test was performed by inserting a standard sterile Schirmer tear test strip (Tear Strips, Biovision Limited, Dunstable, UK, lot n.T197, exp. date: 2021-06) in the inferior conjunctival fornix at the lateral third of the lower eyelid, ensuring contact with the corneal surface, for 1 min in each eye, and baseline values were recorded. All animals were sedated using intramuscular (IM) dexmedetomidine at a dosage of 4 µg/kg. Before sedation, all dogs were kept fasting for 8 h, while water was available ad libitum until 30 min before starting the procedure. Time under sedation for each patient varied from 20 to 45 min depending on the time spent for radiologic study. Following the initial dose of anesthesia, the dogs were unconscious for an average of 30 min. The unconscious patients were positioned in sternal or dorsal recumbency. During the sedation, heart rate (HR), cardiac rhythm, respiratory rate (fR) and rectal temperature (RT) were recorded. The HR was measured using a stethoscope, and the cardiac rhythm was obtained by electrocardiography using lead II. The respiratory rate (fR) was counted from thoracic excursions. The rectal temperature (RT) was measured using a digital thermometer. Dexmedetomidine was not reversed at the end of radiologic study. 

In all animals, tear production was measured 15 min before sedation (T0) and 20 min (T20), 1 h (T1), 2 h (T2), 4 h (T4) and 8 h (T8) after drug administration, in right and left eyes. Only the T20 data point was taken while the dogs were unconscious; during the other data points, all dogs were conscious. In all dogs, the same operator performed the STT-1 readings at each data point, between 8:00 and 16:00 h. After the last STT-1 reading, before the discharge, each dog underwent an ocular exam to evaluate the corneal integrity, including slit-lamp biomicroscopy and fluorescein staining of both eyes.

### 2.2. Statistical Analysis

Statistical analysis was performed by using the calculation software Prism 7.0 (Graph Pad Software, San Diego, CA, USA). The Kolmogorov–Smirnov method was applied to verify their normal distribution. The paired Student’s t-test was applied to evaluate statistical differences between left and right eyes. Due to the absence of differences between the two eyes, data were treated as replicated measures to statistical analysis, according to Ghaffari et al. [38]. 

Tear readings (mm/min) were compared at various data points, using one-way for repeated measures analysis of variance (ANOVA), followed by the post hoc Bonferroni test. *p*-values < 0.05 were considered statistically significant.

## 3. Results

Enrolled dogs were seven males and three females, with age ranging between 14 months and 5 years and body weight of 16 ± 3 kg (mean ± standard deviation (SD)).

**Figure 1:**
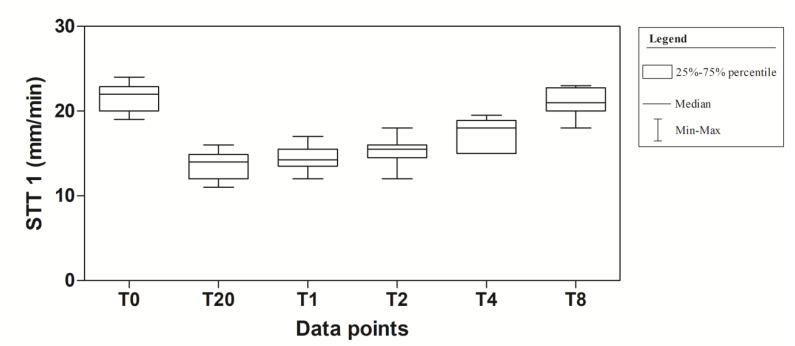
Descriptive statistic (Median, Minimum (Min), Maximum (Max), 25% and 75% Percentiles) of tear production (mm/min) of 10 dogs (*n* = 20 eyes) subjected to dexmedetomidine sedation.

Figure 1 shows the STT-1 readings at the different data points of the experimental protocol.

**Figure 2:**
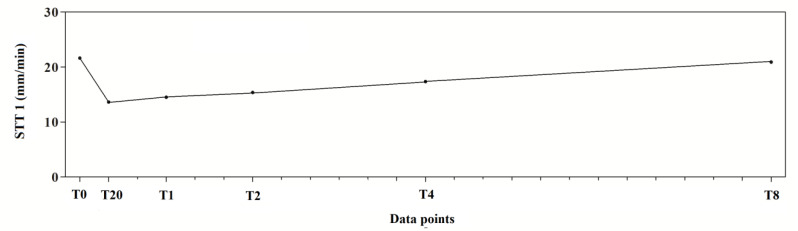
Trend of tear production measured with Schirmer Tear Test 1 (STT-1) in 10 dogs (*n* = 20 eyes) subjected to dexmedetomidine sedation.

The application of one-way for repeated measures ANOVA showed a significant effect of time (F_(5,95)_ = 100.8; *p* < 0.0001). In particular, a significant decrease in tear production at T20, T1, T2 and T4 compared to T0 was observed in the experimental protocol. Dexmedetomidine caused the highest reduction at T20 (40.9%). At T2, the values began to return to the basal level that was reached at T8 (Figure 2). The mean values ± standard deviation (SD) of tear production, expressed in mm/min, observed in the experimental protocol, together with their statistical significance, are showed in Table 1. 

No dogs showed corneal lesions during the experimental period.

## 4. Discussion

Dexmedetomidine is the active optical enantiomer isolated from the racemic compound medetomidine. Alpha2-adrenergic agonists have been used in veterinary anesthesia since the late 1960s, and when in the 1990s dexmedetomidine was developed to reduce the side effects of medetomidine, its use became routine in both human and veterinary medicine [34,35,37]. In dogs and cats, dexmedetomidine produces dose-dependent levels of sedation and the intensity of these effects is similar to that produced by twice the dose of medetomidine [39].

Many studies have already evaluated the effect of α2-agonist medetomidine alone or in combination with other sedatives on tear production in dogs. The authors showed that the tear flow returns to normal values in a short time after reversal of medetomidine with atipamezole; without antagonism, the reduced tear secretion lasted until 1 or 2 h in a dose-dependent manner [17,40,41].

Our results showed that the DEX sedation protocol significantly affected tear production in dogs, in accordance to the only study that assessed the effects of a dexmedetomidine-butorphanol combination on tear production [18]. Even if authors showed that STT-1 values were lower than basal values after 15 min from the reversal of dexmedetomidine, they did not continue to monitor tear secretion. In the present study, the monitoring period for tear secretion was prolonged up to 8 h after drug administration, demonstrating that the decrease in canine tear flow induced by DEX without antagonism lasted up to more than 4 h after sedation.

In the present study, STT-1 values never reach below 10 mm/min. This finding can be related to the low dose of DEX employed, as reported by Kanda et al [41]. However, STT-1 values did not return to the reference range despite recovery from sedation.

The decrease in tear flow induced by dexmedetomidine may be attributed to some mechanisms previously reported, such as central effects, tear gland vasoconstriction, metabolic alterations and nociception [42,43]. The first mechanism may be a direct effect of the central regulation performed by the autonomic nervous system. In particular, the lacrimal gland is innervated by the lacrimal nerve, a branch of the trigeminal nerve, possessing both parasympathetic (cholinergic) and sympathetic (adrenergic) components, suggesting a possible role of both nervous systems in its function. The α2-agonist sedatives, such as dexmedetomidine, are noted to inhibit the sympathetic system. Postsynaptic activation of α2-adrenoceptors in the central nervous system might have decreased basal tear production in previous studies on dogs [37]. α2-agonist induces systemic vasoconstriction, lower heart rate and decreased cardiac output in dogs. Consequently, another mechanism of interference on tear production by dexmedetomidine can be linked to reduced lacrimal gland perfusion and ability to secrete tears. Alteration of metabolism at the cellular level of the lacrimal gland through the α2-adrenoceptor has also been proposed to decrease tear production. Moreover, it was also suggested that increased antinociception modulated by α2-adrenoceptor decreases reflex tear secretion [42].

Sedation or general anesthesia can determine the perioperative dry eye syndrome, also known as exposure keratopathy (EK), as an ocular complication encountered in both human and veterinary medicine [8,17,22]. It is associated with the instability and hyperosmolarity of tear film and in severe cases, causes inflammation, neurosensory damage of the ocular surface and consequent development of permanent focal lesions of the cornea. If small perioperative corneal abrasions heal quickly due to the normal regeneration of corneal epithelium in 24–72 h, sometimes, the keratopathy becomes clinically apparent in a few days or weeks post-sedation with the risk to chronicity [8].

Although in our study no corneal ulcers were diagnosed during the experimental period, the appearance of corneal degenerative changes in the days following sedation cannot be excluded.

It is important for clinicians to know the tear production effects of dexmedetomidine, when used alone. They should observe the ocular surface during anesthesia and after recovery from anesthesia, treating the canine eyes with tear substitutes to protect ocular surface health and the welfare of dogs. The ocular lubrication should be performed not only during the sedation but at least up to 12 h after the treatment.

The stability of tear fluid is affected by whole-body hydration status. Modest changes of hydration status can cause tear fluid osmolarity disorders, leading to surface damage in aqueous deficient and evaporative dry eyes [2]. Aquaporin channels are responsible for fluid movements into and out of the eye. In the present study, dogs with clinical evidence of altered hydration status and abnormal values of complete blood count (CBC) and biochemical parameters were excluded to avoid the interference of the state of hydration on the tear fluid production.

Future studies on AQP expression would be interesting to investigate their role in canine ophthalmic diseases related to changes in hydration status and to alterations of tear fluid composition, such as those that happen during sedation.

Currently, there is increased interest in the use of DEX as an agent for sedation in non-intubated human patients before or during surgical and other procedures. In the European Union, DEX was approved for the sedation of adult patients; conversely, the use of DEX in pediatric anesthesia is as yet “off-label”, despite a strong evidence base for its use [34,35,44]. In humans, the occurrence of exposure keratopathy post-sedation becomes a concern in the intensive care unit (ICU), where the admitted patients need to be maintained for a long time under sedation or invasive mechanical ventilation, being exposed to low temperature and humidity, which contribute to the occurrence of eye dryness [13,39,44].

The closer resemblances in ocular physiology between dogs and humans can justify the integration of companion dogs into preclinical studies rather than traditional laboratory animals such as rabbits, mice and rats, as reported in the literature [45,46,47,48,49,50,51,52,53,54,55,56,57,58,59].

## 5. Conclusions

This study confirmed the reduction of the tear production by sedative drugs, such as dexmedetomidine, which exerts its action up to more than 4 h after sedation and underlines the necessity to use artificial moisturizing lubricant eye drops to reduce the corneal discomfort. Certainly, further studies on tear fluid dynamics during anesthesia are needed to better understand the clinical and pathologic patterns of ocular surface diseases.

## Figures and Tables

**Table 1:** Mean values ± standard deviation (SD) of tear secretion recorded using STT-1, expressed in its conventional unit (mm/min) in all data points investigated, together with their statistical differences.

	Data Points					
	T0	T20	T1	T2	T4	T8
**Mean ± SD**	21.63 ± 1.43	13.63 ± 1.43	14.52 ± 1.37	15.38 ± 1.41	17.35 ± 1.69	20.70 ± 1.89
**T0**		0.0001	0.0001	0.0001	0.0001	NS
**T20**	0.0001		NS	0.001	0.0001	0.0001
**T1**	0.0001	NS		NS	0.0001	0.0001
**T2**	0.0001	0.001	NS		0.001	0.0001
**T4**	0.0001	0.0001	0.0001	0.001		0.0001
**T8**	NS	0.0001	0.0001	0.0001	0.0001	

NS: no significant.

## Data Availability

The data presented in this study are available on request from the corresponding author.
